# Poor prognosis of NSCLC located in lower lobe is partly mediated by lower frequency of EGFR mutations

**DOI:** 10.1038/s41598-020-71996-7

**Published:** 2020-09-10

**Authors:** Hyun Woo Lee, Young Sik Park, Sangshin Park, Chang-Hoon Lee

**Affiliations:** 1 Division of Pulmonary and Critical Care Medicine, Department of Internal Medicine, Seoul Metropolitan Government-Seoul National University Boramae Medical Center, Seoul, Republic of Korea; 2 Division of Pulmonary and Critical Care Medicine, Department of Internal Medicine, Seoul National University College of Medicine, Seoul National University Hospital, Seoul, Republic of Korea; 3 Department of Pediatrics, Center for International Health Research, Rhode Island Hospital, The Warren Alpert Medical School of Brown University, Providence, RI USA; 4 Graduate School of Urban Public Health, University of Seoul, Seoul, Republic of Korea

**Keywords:** Cancer epigenetics, Outcomes research

## Abstract

It is controversial whether a tumor located in the lower lobe is related with worse outcome of non-small cell lung cancer (NSCLC). This study aimed to clarify the prognostic role of primary tumor location in NSCLC. Patients newly diagnosed with NSCLC in a tertiary referral hospital from January 2011 to December 2014 were followed up for 5 years. Of the 2,289 NSCLC cases, 911 (39.8%) cases pertained to lower lobe cancers. Patients with lower lobe cancer showed a higher all-cause mortality rate than those with non-lower lobe cancer (48.6% vs. 40.3%, *p* < 0.001). Patients with lower lobe cancer had a lower proportion of adenocarcinoma histology and epidermal growth factor receptor (EGFR) mutations. Furthermore, compared to patients with non-lower lobe cancer, those with lower lobe cancer had a higher level of tumor markers (neuron-specific enolase and cytokeratin fragment 21-1). Mediation analysis revealed that the association between lower lobe cancer and higher all-cause mortality could be explained by an indirect pathway through EGFR mutations (percent mediated = 17.3%, *p* = 0.005). The sensitivity analysis for adenocarcinoma patients showed similar results (percent mediated = 18.8%, *p* = 0.021). Lower lobe cancer is associated with a higher all-cause mortality risk in patients with NSCLC, which is partly mediated by a lower proportion of EGFR mutations.

## Introduction

Lung cancer is the second most commonly detected cancer and the most leading cause of cancer death in the world, although incidence and mortality have been decreased in recent decades^1^. There have been advances in early detection and standard treatment for lung cancer, but the 5-year survival rate is still 4–55% according to localized or advanced stage^2^. Lung cancer is a heterogeneous disease with different clinicopathological features, and identifying subtypes by molecular abnormalities and biomarkers is mandatory for accurate prediction of treatment success and clinical prognosis. Exploring the differences in prognosis according to the lung cancer phenotypes is a fundamental step for elucidating the role of biologic markers.

The location of non-small cell lung cancer (NSCLC) is considered as an important factor in predicting treatment efficacy and clinical prognosis. Several studies have shown that the operation site or side influence the treatment outcome^3^. The location of NSCLC is related with the distribution of lymph node (LN) metastasis^4,5^. The unexpected upstaging by surgical LN evaluation is more frequently found in the lower lobe^6^. Differences of histologic type^7,8^ and epidermal growth factor receptor (EGFR) mutations^9^ were found according to tumor location. However, it has not been clearly explained how tumor location relates to clinical prognosis. In addition, it is unclear whether lower lobe cancer is significantly associated with worse prognosis. Several studies have revealed that the tumors located in non-upper lobes had poorer clinical outcomes in NSCLC with resectable stages^10,11^; contrasting results have been reported in some studies^12,13^.

We aimed to investigate whether a tumor located in the lower lobe is associated with higher mortality risk and to identify a plausible mediator between the tumor location and mortality in patients with NSCLC.

## Methods

We confirmed that all methods were carried out in accordance with the guidelines and regulations for strengthening the reporting of observational studies in epidemiology (STROBE) statement^14^.

### Study design and setting

This retrospective cohort study was conducted by reviewing the electronic medical records of patients newly diagnosed with NSCLC from January 2011 to December 2014 and followed up for 5 years at a tertiary teaching hospital in South Korea. After NSCLC diagnosis, the treatment plan was made by multidisciplinary discussion (MDD). Mortality data were obtained from the Ministry of Interior and Safety of Korea. Overall, the survival rate was assessed from the date of the diagnosis to the date of death or the last follow-up date.

### Participants

During the study period, patients with pathologically proven NSCLC were recruited. Chest computed tomography (CT) at the initial stage of diagnosis was used to evaluate the location of the primary tumor (lower lobe or non-lower lobe). Cases in which the primary tumor location was difficult to identify because of multiple lesions or tumors involving two or more lobes were excluded. Non-lower lobes included the right upper lobe, the left upper lobe, and the right middle lobe. EGFR mutations and tumor markers such as neuron-specific enolase (NSE), cytokeratin fragment (CYFRA) 21-1 were conducted by the clinicians’ decision as a routine practice.

### Variables and measures

The demographic information included age; sex, body mass index (BMI); smoking status; Eastern Cooperative Oncology Group (ECOG) performance status; presence of respiratory symptoms; pulmonary function test; pathology; tumor, node, metastases (TNM) stage; and initial treatments. Pulmonary function test included forced expiratory volume in one second (FEV1), forced vital capacity (FVC), and the FEV1/FVC%. In our study population, a positron emission tomography-computed tomography (PET/CT) and a magnetic resonance imaging (MRI) of the brain was performed in most patients for clinical staging. An endobronchial ultrasound-guided transbronchial needle aspiration was conducted to evaluate mediastinal LN status if it was clinically indicated. The new 8th TNM staging system was applied to the clinical and pathologic stages. For purposes of accurate staging from I to IV, we combined clinical and pathologic staging. The pathologic stage was used in patients who underwent surgery, while the clinical stage was used on the remaining patients. Also, we verified if the TNM stage changed after surgery. Active treatment was defined as surgical resection, radiotherapy, and chemotherapy for curative purposes or for palliative care. The location of the primary tumor was classified into lower lobe versus non-lower lobe based on a chest CT. Information on the variables known to be prognostic factors was reviewed and included age, sex, BMI, smoking status, performance status, symptoms at the moment of diagnosis, lung function, histology, standardized uptake value (SUV) of the main mass, tumor markers (NSE, CYFRA 21-1, and carcinoembryonic antigen [CEA]), EGFR mutations, and anaplastic lymphoma kinase (ALK) translocation. The mediator candidates were determined among these variables considering differences between the lower lobe and non-lower lobe cancer. The outcomes were all-cause mortality and time to all-cause death.

### Statistical methods

The chi-square test was used for categorical variables and the student *t*-test was used for continuous variables. The Kaplan–Meier method was used to compare the time to all-cause mortality between non-lower and the lower lobe cancer groups, and the difference was estimated by the log-rank test. A multivariable Cox proportional hazard assumption test was performed with model 1 and 2. Model 1 included covariates except for mediator candidates. Model 2 included the mediator candidates in addition to the covariates for model 1. We performed mediation analysis only for mediators (EGFR, NSE, CYFRA, or adenocarcinoma) that met the following requirements^15^: (1) the significant relationship between tumor location and the mediator; (2) the significant relationship between the mediator and mortality; (3) the significant relationship between tumor location and mortality in the absence of the mediator; and (4) the attenuated relationship between tumor location and mortality when the mediator was included in the model. Percent mediated was calculated as the ratio of the absolute value of the indirect effect to the absolute value of the total effect of metabolic components on the outcome^16^. *P* < 0.05 was considered significant difference. All the statistical analyses were performed using the Stata statistical software version 14.2 (StataCorp LP, College Station, TX, USA) and SAS 9.4 (SAS Institute, Cary, NC).

### Ethics

This study was approved by the Institutional Review Board of Seoul National University Hospital (H-1611-047-807). Informed consent was waived.

## Results

**Figure 1:**
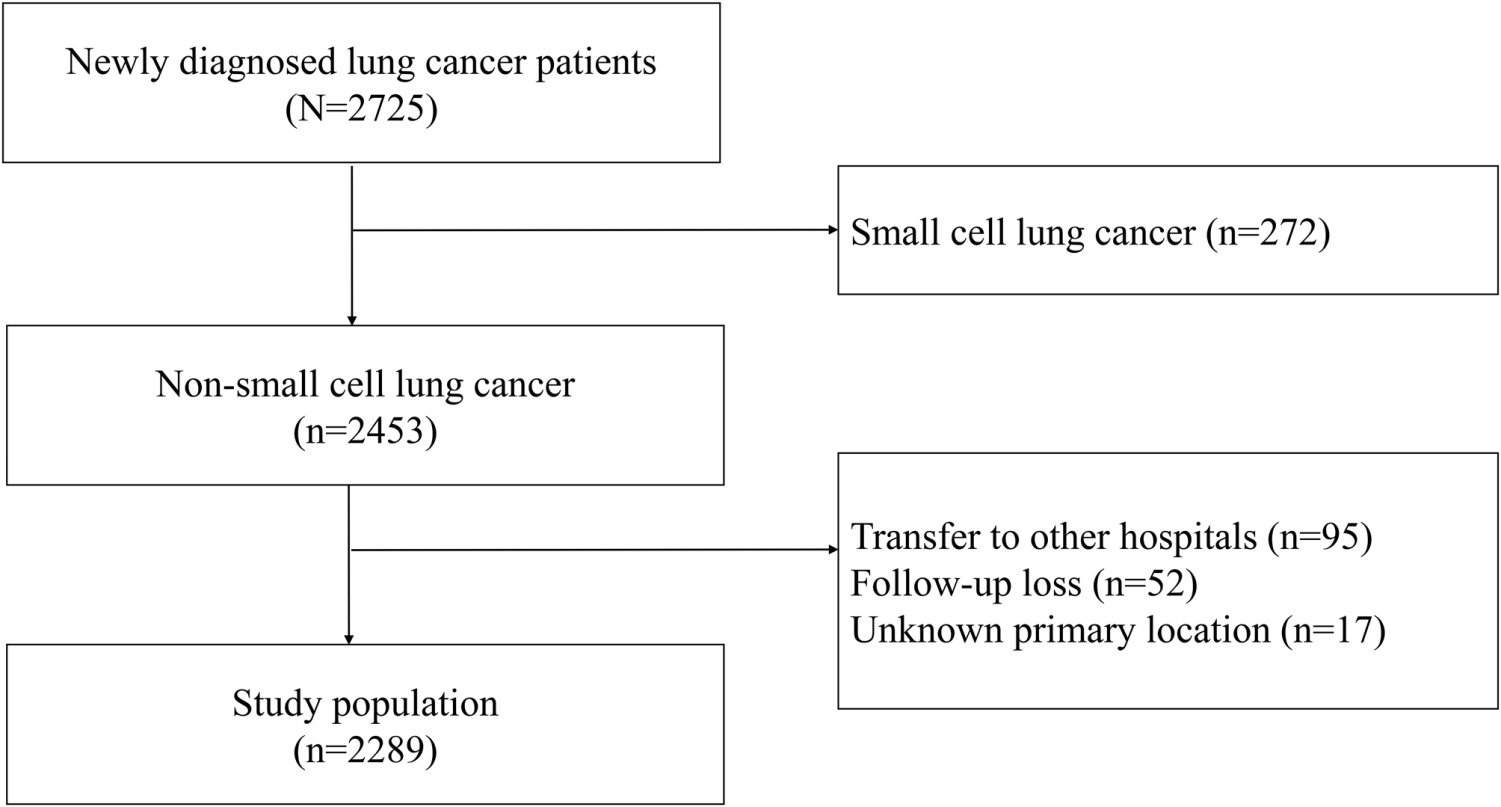
Flow diagram of study population.

A total of 2,453 patients were diagnosed with NSCLC from January 2011 to December 2015. Of them, we excluded patients with small cell lung cancer and those who were transferred to other hospitals after the initial diagnosis or those who were lost to follow-up and whose primary location could not be assessed. Finally, 2,289 patients were included in our study (Fig. 1). Among them, 1,378 (60.2%) had a primary tumor located in non-lower lobes, while 911 (39.8%) had a primary tumor in the lower lobes. During mean 3.5(± 1.9) years of observation, we found 999 (43.6%) were died. The patients with NSCLC located in the lower lobes had a higher all-cause mortality rate than those with non-lower lobe cancers (48.6% and 40.3% respectively, *P* < 0.001).

### Patients characteristics

The baseline characteristics were described according to the tumor location in Table 1. There were 911 patients (39.8%) with primary tumors in the lower lobes. There was no difference in age, sex, BMI, smoking status, ECOG performance status, accompanying symptoms, and pulmonary function test between the non-lower and the lower lobe group. We found no significant differences in lung cancer TNM stage and the SUV of the main mass. Active treatments were performed at a similar rate in both groups.

**Table 1:** Baseline characteristics according to tumor location of non-small cell lung cancer patients.

Variable	Non-lower lobe (n = 1,378)	Lower lobe (n = 911)	*P* value
**Age, % (n = 2,289)**			0.270
< 40 year	21 (1.5%)	14 (1.5%)	
40–59 year	339 (24.6%)	207 (22.7%)	
60–79 year	936 (67.9%)	618 (67.8%)	
≥ 80 year	82 (6.0%)	72 (7.9%)	
Male, % (n = 2,289)	502 (36.4%)	329 (36.1%)	0.913
**Degree of obesity, % (n = 2,289)**			0.873
Underweight, BMI < 18.5 kg/m^2^	82 (6.0%)	54 (5.9%)	
Normal, BMI = 18.5–22.9 kg/m^2^	539 (39.1%)	359 (39.5%)	
Overweight, BMI = 23.0–24.9 kg/m^2^	353 (25.6%)	220 (24.2%)	
Obese, BMI ≥ 25.0 kg/m^2^	404 (29.3%)	277 (30.4%)	
**Smoking status, % (n = 2,289)**			0.865
Ever smoking	847 (61.5%)	564 (61.9%)	
**ECOG,% (n = 2,289)**			0.108
0	641 (46.5%)	380 (41.7%)	
1	590 (42.8%)	412 (45.2%)	
2	113 (8.2%)	93 (10.2%)	
3	30 (2.2%)	25 (2.7%)	
4	4 (0.3%)	1 (0.1%)	
Respiratory symptoms at diagnosis, % (n = 2,289)	679 (49.3%)	466 (51.2%)	0.403
**Pulmonary function test (n = 2,028)**			
FEV_1_% of predicted	98.0 ± 22.6	97.7 ± 22.7	0.760
FVC % of predicted	97.1 ± 16.9	95.9 ± 17.5	0.128
FEV_1_/FVC %	71.1 ± 11.6	71.3 ± 11.2	0.745
**Pathology, % (n = 2,289)**			0.011
Adenocarcinoma	922 (66.9%)	554 (60.8%)	
Squamous cell carcinoma	326 (23.7%)	260 (28.5%)	
Others	130 (9.4%)	97 (10.6%)	
**Stage, % (n = 2,289)**			0.133
I	440 (31.9%)	274 (30.1%)	
II	145 (10.5%)	112 (12.3%)	
III	326 (23.7%)	189 (20.7%)	
IV	467 (33.9%)	336 (36.9%)	
SUV of main mass (n = 2,063)	12.2 ± 7.2	11.7 ± 7.4	0.099
EBUS-TBNA (n = 2,289)	371 (26.9%)	267 (29.3%)	0.231
**Tumor markers**			
NSE, ng/mL (n = 1,059)	21.7 ± 16.7	26.2 ± 31.7	0.003
CEA, ng/mL (n = 1,962)	54.4 ± 385.7	34.7 ± 181.2	0.182
CYFRA 21-1, ng/mL (n = 1,740)	6.0 ± 17.4	7.9 ± 17.1	0.026
**EGFR mutations, % (n = 1,672)**	438 (42.6%)	221 (34.4%)	0.001
Exon 18, %	23 (2.2%)	11 (1.7%)	0.569
Exon 19, %	193 (18.7%)	120 (18.7%)	1.000
Exon 20, %	22 (2.1%)	12 (1.9%)	0.831
Exon 21, %	214 (20.8%)	86 (13.4%)	< 0.001
ALK translocation, % (n = 1,678)	46 (4.5%)	39 (6.0%)	0.199
Active treatment, % (n = 2,289)	1,309 (95.0%)	855 (93.9%)	0.280

*ALK* anaplastic lymphoma kinase; *BMI* body mass index; *CEA* carcinoembryonic antigen; *CYFRA* cytokeratin fragment; *EBUS-TBNA* endobronchial ultrasound-guided transbronchial needle aspirate; *ECOG* Eastern Cooperative Oncology Group; *EGFR* epidermal growth factor receptor; *FEV1* forced expiratory volume in 1 second; *FVC* forced vital capacity; *NSE* neuron-specific enolase; *SUV* standardized uptake value.

In pathology, adenocarcinomas are more frequently found in the non-lower lobe group, while squamous-cell carcinomas are more likely to be detected in the lower lobe group. Tumor markers such as NSE and CYFRA 21-1 were elevated in the lower lobe group. EGFR mutations were more frequently detected in the non-lower lobe group. Notably, we found that exon 21 mutations significantly contributed to the difference of EGFR mutation frequency between the non-lower lobe and the lower lobe groups (20.8% and 13.4% respectively; *P* < 0.001).

### Comparison of survival rate between the non-lower lobe and the lower lobe groups

**Figure 2:**
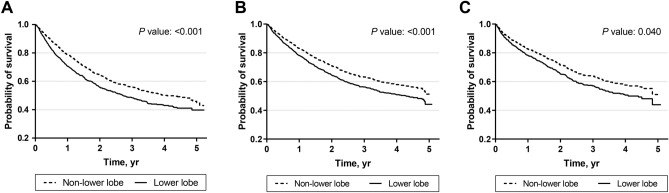
Kaplan–Meier survival curve with univariate model and multivariate Cox proportional hazard models. (**A**) Kaplan–Meier survival curve with univariate model; (**B**) Cox proportional hazard model 1 with the covariates except for the mediator candidates; (**C**) Cox proportional hazard model 2 with the covariates including the mediator candidates. The covariates included age, sex, smoking status, performance status, presence of symptoms, body mass index, standardized uptake value of main mass, stage, anaplastic lymphoma kinase translocation, and active treatment. The mediator candidates included adenocarcinoma histology, serum neuron-specific enolase level, serum cytokeratin fragment level, and epidermal growth factor receptor mutations.

Covariates that had significant relationships with all-cause mortality were: pathology, EGFR mutations, serum NSE, and serum CYFRA 21-1 (Supplementary information 1). We determined these covariates as the mediator candidates. In the unadjusted Kaplan–Meier curve, a higher risk of all-cause mortality was observed in the lower lobe group than in the non-lower lobe group (*P* < 0.001, Fig. 2A). Similar results were found in the Kaplan–Meier curves adjusted by the covariates (Fig. 2B) and the mediator candidates (Fig. 2C). Multivariable Cox regression analysis in model 1 showed that the lower lobe cancer was associated with a higher risk of all-cause mortality (HR 1.34, 95% *CI* 1.14–1.59, *P* = 0.001) (Table 2). In addition, higher age (≥ 60), ever smoking, ECOG performance status ≥ 2, accompanying symptoms, higher SUV of the main mass ≥ 11.2, and higher stage were all related with a higher risk of all-cause mortality. In contrast, ALK translocation and active treatment were associated with a lower risk of all-cause mortality (Table 2). In model 2, multivariable analyses with the Cox proportional hazards regression model revealed that lower lobe cancer was associated with a higher risk of all-cause mortality (HR 1.31, 95% *CI* 1.01–1.70, *P* = 0.040). Also, NSE ≥ 16.3 ng/mL and CYFRA 21-1 ≥ 3.3 ng/mL increased the risk of all-cause mortality, while EGFR mutations decreased the risk of all-cause mortality. Sensitivity analysis with the patients who were diagnosed with adenocarcinomas showed similar results (Supplementary information 2).

**Table 2:** Multivariable Cox proportional hazard model analysis according to tumor location.

Variable	Model 1			Model 2		
	HR	95% CI	*P* value	HR	95% CI	*P* value
Tumor location, lower lobe	1.34	1.14–1.59	0.001	1.31	1.01–1.70	0.039
Age ≥ 60 year	1.72	1.40–2.12	< 0.001	1.63	1.19–2.24	0.003
Sex, male	0.82	0.62–1.10	0.18	1.33	0.84–2.11	0.23
Ever smoking	1.45	1.09–1.93	0.011	1.58	0.98–2.55	0.06
ECOG ≥ 2	2.25	1.76–2.87	< 0.001	2.00	1.39–2.89	< 0.001
Presence of symptoms	1.41	1.17–1.70	< 0.001	1.24	0.94–1.63	0.13
BMI: 23.0–24.9 kg/m^2^	0.86	0.70–1.05	0.14	0.84	0.62–1.15	0.29
BMI: ≥ 25.0 kg/m^2^	0.88	0.72–1.07	0.19	0.79	0.58–1.08	0.14
SUV of main mass ≥ 11.2	1.41	1.19–1.69	< 0.001	1.31	1.00–1.71	0.050
Stage II	1.47	0.97–2.25	0.07	1.21	0.65–2.26	0.54
Stage III	3.36	2.40–4.71	< 0.001	3.40	2.06–5.61	< 0.001
Stage IV	8.98	6.64–12.15	< 0.001	9.01	5.64–14.38	< 0.001
ALK translocation	0.42	0.27–0.66	< 0.001	0.27	0.13–0.56	< 0.001
Active treatment	0.33	0.23–0.47	< 0.001	0.46	0.28–0.77	0.003
Adenocarcinoma				1.06	0.74–1.52	0.74
NSE ≥ 16.3 ng/mL				1.42	1.09–1.85	0.009
CYFRA ≥ 3.3 ng/mL				1.55	1.17–2.06	0.002
EGFR mutations				0.46	0.33–0.63	< 0.001

Possible mediational factors (adenocarcinoma, NSE, CYFRA 21-1, EGFR) were excluded in analysis with model 1 and included in analysis with model 2.

*ALK* anaplastic lymphoma kinase; *BMI* body mass index; *CYFRA* cytokeratin fragment; *NSE* neuron-specific enolase; *SUV* standardized uptake value.

### Stage change after complete surgical resection

Among the 2,289 patients, 1,072 (46.8%) underwent a complete surgical resection. TNM stage changed in 498 patients (46.5%) after surgery; pathologic upstage happened in 369 patients (34.4%), and downstaging happened in 129 patients (12.0%). The proportion of stage change from clinical to pathologic was not significantly different between the non-lower lobe and the lower lobe groups (Table 3).

**Table 3:** Stage shift from clinical to pathologic stage in the patients who underwent complete resection.

	Non-lower lobe (n = 652)	Lower lobe (n = 420)	*P* value
Upstage	230 (35.3%)	139 (33.1%)	0.504
No change	348 (53.4%)	226 (53.8%)	0.939
Downstage	74 (11.4%)	55 (13.1%)	0.446

### Causal mediation analysis

**Figure 3:**
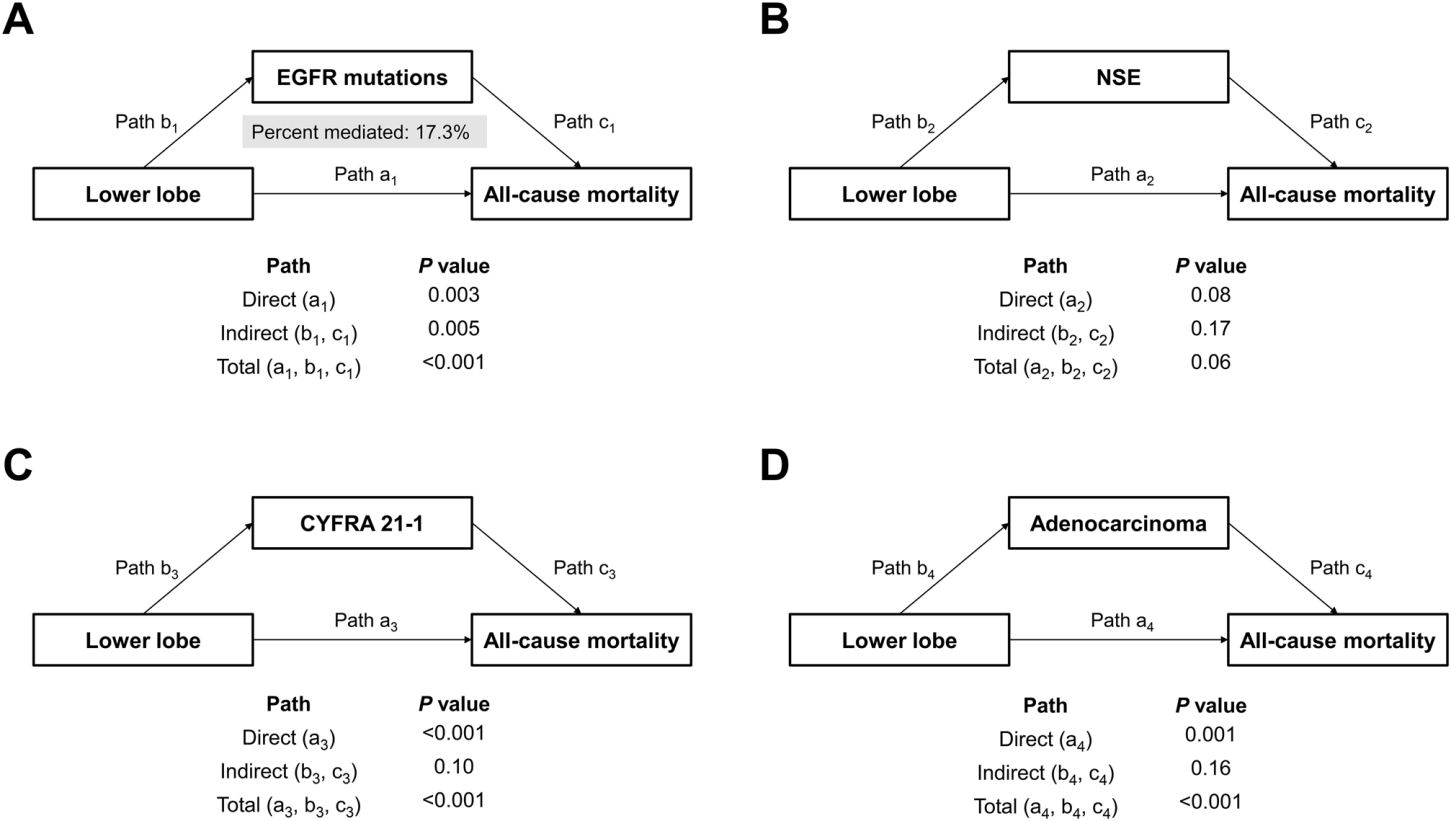
Causal mediation analysis in non-small cell lung cancer patients. (**A**) Mediation analysis for indirect effect of EGFR mutations; (**B**) Mediation analysis for indirect effect of NSE; (**C**) Mediation analysis for indirect effect of CYFRA; (**D**) Mediation analysis for indirect effect of adenocarcinoma. CYFRA, cytokeratin fragment; EGFR, epidermal growth factor receptor mutations; NSE, neuron-specific enolase.

In mediation analysis to identify causal associations between the tumor location and survival, EGFR mutations showed a statistically significant indirect effect (*P* = 0.005, Fig. 3). In the association between lower lobe location and higher mortality risk, 17.3% could be explained by lower expression of EGFR mutations. The sensitivity analysis of the patients diagnosed with adenocarcinomas showed that the percent of association mediated was 18.8% through EGFR mutations alone, and this indirect association was statistically significant (*P* = 0.021, Supplementary information 3).

## Discussion

In the present study, the patients with NSCLC in the lower lobes had a higher risk of all-cause mortality than those with non-lower lobe cancer. The patients with lower lobe cancer had a higher proportion of non-adenocarcinoma histology, a higher tumor marker level, and a lower proportion of EGFR mutations, which were also associated with an increased risk for 5-year all-cause mortality. In our knowledge, this is the first study that evaluated the relationship between lung cancer location and prognosis including patients with unresectable stage. Because of more permittable inclusion criterion in terms of lung cancer stage compared to previously published studies^11,13,17,18^, the 5-year survival rate was lower in our study subjects (44% vs. 62–74%). We found that lower lobe location and a lower expression of EGFR mutations were the independent factors linked to poor prognosis regardless of important clinical factors including lung cancer stage. In the mediation analysis, a significant indirect pathway through EGFR mutations in the relationship between the lower lobe location and all-cause mortality was observed. In the sensitivity analysis for adenocarcinoma patients, EGFR mutations were also identified as a significant mediator. These findings suggest that the lower frequency of EGFR mutations can partly mediate the higher all-cause mortality risk in the lower lobe NSCLC.

The prognostic role of the lobar location in NSCLC has not been well validated. In the early 2000s, two Japanese groups reported that the upper lobe location of a primary tumor allowed for better survival in patients with a completely resected stage IIIA^19,20^. In 2007, Ou et al. suggested that the non-upper lobe location was a risk factor for stage I patients^10^. There have been several efforts to determine why NSCLCs in lower lobes pose a worse prognosis when compared to those in the non-lower lobe. First, accurate clinical staging remains a challenge, especially in lower lobe cancers. A prospective study showed stage I or II NSCLCs located in the lower lobes were more likely to be upstaged in histologic diagnosis when compared to those in the upper lobes^6^. The main reason for stage misclassification in lower lobe cancers was a more advanced tumor (T) stage attributed by a radiologically uncertain pleural or chest wall invasion and an unsuspected spread to central airway or mediastinum. Second, the effectiveness of treatments may be different according to tumor location. Worse treatment outcomes for radiation therapy were reported in patients with lower lobe cancers^17,21^. The majority of the lower lobe cancers were not good candidates for radiation therapy than the non-lower lobe cancers, because there are more obstacles such as heart during the radiation treatment. Third, the predisposing location of underlying chronic lung disease may influence the prognosis according to the location of NSCLC. For example, idiopathic pulmonary fibrosis is frequently detected in lower lobes and is also associated with worse prognosis of NSCLC^22^. Fourth, EGFR mutations could be the link between tumor location and prognosis. EGFR mutations are less likely to be detected in the lower lobe cancers^9^. Considering that EGFR mutation is a favorable predictive marker^23^, lower lobe cancers are expected to have poor prognosis than non-lower lobe cancers. Therefore, our interest was to prove whether the relationship between lower lobe location and prognosis can be explained by expression of EGFR mutations in NSCLC.

EGFR mutation has been studied as a favorable prognostic marker in NSCLC. In the post-hoc analysis of phase III randomized controlled trial, EGFR mutations were related to a better survival rate, irrespective of treatment^24^. There is a higher rate of EGFR mutations in Asians^25^. In a large study, in which Asians were not included, scientists did not find a significant relationship between tumor location and clinical prognosis^13^. One plausible reason for inconsistent results about the prognostic role of cancer location is the different proportion of multiple EGFR mutations^23^. In our analyses on various EGFR mutations, exon 19 and 21 mutations were significantly related with survival, while exon 18 and 20 mutations were not. However, it is still unclear whether the differences in genetic abnormalities of the study population are the main reason for the difference in prognosis.

Our study has certain strengths. First, to our knowledge, this was the first mediation analysis study exploring why the survival difference was observed according to primary tumor location. Our results validate the reason why previous studies have shown similar outcomes. Second, we analyzed a large population with accurate lung cancer stage. In this study population, radiologic or interventional work-ups for lung cancer staging were fully available and determined by MDD. Similarly, covariates were evenly distributed according to the non-lower lobe and the lower lobe group, except for the mediator candidates. Sufficient patient data were available for the sensitivity analysis for the evaluated patients with lung adenocarcinomas. Third, our study included various prognostic factors as covariates to adjust for the association between tumor location and prognosis. In particular, our study was different in that a serum level of tumor markers was also assessed with clinicopathological features. Increased levels of NSE have also been reported in NSCLCs and reflects neuroendocrine components^26^. CYFRA 21-1 is highly expressed by all epithelial cells and represents a useful indicator of epithelial differentiation^26^. NSE and CYFRA 21-1 have been reported as predictive factors of clinical prognosis in NSCLC patients^27,28^.

In conclusion, our study showed that a lower lobe cancer is associated with a higher all-cause mortality risk in patients with NSCLC, which is partly mediated by a lower proportion of EGFR mutations in lower lobe cancers.

## Supplementary information

Supplementary Information 1.

Supplementary Information 2.

Supplementary Information 3.

## Abbreviations

ALK: Anaplastic lymphoma kinase
CEA: Carcinoembryonic antigen
CT: Computed tomography
CYFRA: Cytokeratin fragment
EGFR: Epidermal growth factor receptor
FEV1: Forced expiratory volume in 1 second
FVC: Forced vital capacity
MDD: Multidisciplinary discussion
NSCLC: Non-small cell lung cancer
NSE: Neuron-specific enolase
PET: Positron emission tomography
SUV: Standardized uptake value
