# Supplementary material for: Poor prognosis of NSCLC located in lower lobe is partly mediated by lower frequency of EGFR mutations
Source: Sci Rep. 2020 Sep 10;10:14933. doi: 10.1038/s41598-020-71996-7 (PMC7483476; doi:10.1038/s41598-020-71996-7)
Supplement: Supplementary file 1 — Supplementary Information 1. [file 41598_2020_71996_MOESM1_ESM.docx]

**Supplementary information 1. Comparison of covariables according to survivor and non-survivor**

| **Variable** | **Survivor (n=1,290)** | **Non-survivor (n=999)** | ***P* value** |
| --- | --- | --- | --- |
| **Age, % (n=2,289)** |  |  | <0.001 |
| <40 year | 25 (1.9%) | 10 (1.0%) |  |
| 40-59 year | 383 (29.7%) | 163 (16.3%) |  |
| 60-79 year | 836 (64.8%) | 718 (71.9%) |  |
| ≥80 year | 46 (3.6%) | 108 (10.8%) |  |
| **Male, % (n=2,289)** | 559 (43.3%) | 272 (27.2%) | <0.001 |
| **Degree of obesity, % (n=2,289)** |  |  | <0.001 |
| Underweight, BMI<18.5 kg/m^2^ | 50 (3.9%) | 86 (8.6%) |  |
| Normal, BMI=18.5-22.9 kg/m^2^ | 472 (36.6%) | 426 (42.7%) |  |
| Overweight, BMI=23.0-24.9 kg/m^2^ | 327 (25.3%) | 246 (24.6%) |  |
| Obese, BMI≥25.0 kg/m^2^ | 441 (34.2%) | 240 (24.0%) |  |
| **Smoking status, % (n=2,289)** |  |  | <0.001 |
| Ever smoking | 692 (53.6%) | 719 (72.0%) |  |
| **ECOG,% (n=2,289)** |  |  | <0.001 |
| 0 | 752 (58.3%) | 269 (26.9%) |  |
| 1 | 493 (38.2%) | 509 (51.0%) |  |
| 2 | 40 (3.1%) | 166 (16.6%) |  |
| 3 | 4 (0.3%) | 51 (5.1%) |  |
| 4 | 1 (0.1%) | 4 (0.4%) |  |
| **Presence of respiratory symptoms, % (n=2,289)** | 472 (36.6%) | 673 (67.4%) | <0.001 |
| **Pulmonary function test (n=2,028)** |  |  |  |
| FEV1 % of predicted | 102.4±21.4 | 91.3±22.8 | <0.001 |
| FVC % of predicted | 99.8±15.6 | 91.8±18.3 | <0.001 |
| FEV1/FVC % | 72.8±10.6 | 68.9±12.2 | <0.001 |
| **Pathology, % (n=2,289)** |  |  | <0.001 |
| Adenocarcinoma | 935 (72.5%) | 541 (54.2%) |  |
| Squamous cell carcinoma | 266 (20.6%) | 320 (32.0%) |  |
| Others | 89 (6.9%) | 138 (13.8%) |  |
| **Stage, % (n=2,289)** |  |  | <0.001 |
| I | 622 (48.2%) | 92 (9.2%) |  |
| II | 183 (14.2%) | 74 (7.4%) |  |
| III | 271 (21.0%) | 244 (24.4%) |  |
| IV | 214 (16.6%) | 589 (59.0%) |  |
| **Tumor location** |  |  |  |
| Lower lobe | 468 (36.3%) | 443 (44.3%) | <0.001 |
| **SUV of main mass (n=2,063)** | 10.0±6.6 | 14.8±7.3 | <0.001 |
| **EBUS-TBNA (n=2,289)** | 354 (27.4%) | 284 (28.4%) | 0.635 |
| **Tumor markers** |  |  |  |
| NSE, ng/mL (n=1,059) | 20.7±19.2 | 26.4±27.7 | <0.001 |
| CEA, ng/mL (n=1,962) | 16.8±71.9 | 87.4±483.0 | <0.001 |
| CYFRA 21-1, ng/mL (n=1740) | 3.6±4.7 | 11.6±26.1 | <0.001 |
| **EGFR mutations, %** **(n=1,672)** | 477 (45.4%) | 182 (29.3%) | <0.001 |
| Exon 18, % | 18 (1.7%) | 16 (2.6%) | 0.303 |
| Exon 19, % | 236 (22.5%) | 77 (12.4%) | <0.001 |
| Exon 20, % | 20 (1.9%) | 14 (2.3%) | 0.755 |
| Exon 21, % | 217 (20.7%) | 83 (13.3%) | <0.001 |
| **ALK translocation, % (n=1,678)** | 60 (5.7%) | 25 (4.0%) | 0.166 |
| **Active treatment, % (n=2,289)** | 1276 (98.9%) | 888 (88.9%) | <0.001 |

ALK=anaplastic lymphoma kinase; CEA=Carcinoembryonic antigen; CYFRA=cytokeratin fragment; EBUS-TBNA= Endobronchial ultrasound-guided transbronchial needle aspirate; ECOG= Eastern Cooperative Oncology Group; EGFR=epidermal growth factor receptor; FEV1=forced expiratory volume in 1 second; FVC=forced vital capacity; NSE=neuron-specific enolase; SUV=standardized uptake value

**Title: Poor prognosis of NSCLC located in lower lobe is partly mediated by EGFR mutations**

**Running title: prognosis in lower lobe cancer**

**Hyun Woo Lee^1*^, Young Sik Park^2*^**, Sangshin Park^3,4^, Chang-Hoon Lee^2^

^1^ Division of Pulmonary and Critical Care, Department of Internal Medicine, Seoul Metropolitan Government-Seoul National University Boramae Medical Center, Seoul, South Korea.

^2^ Division of Pulmonary and Critical Medicine, Department of Internal Medicine, Seoul National University College of Medicine, Seoul National University Hospital, Seoul, South Korea

^3^ Department of Pediatrics, Center for International Health Research, Rhode Island Hospital, The Warren Alpert Medical School of Brown University, Providence, RI, United States

^4^ Graduate School of Urban Public Health, University of Seoul, Seoul, Republic of Korea

*Two co-first authors were equally contributed to the present work.

**Corresponding author:** Chang-Hoon Lee, M.D., Associate Professor, Division of Pulmonary and Critical Care Medicine, Department of Internal Medicine, Seoul National University Hospital, Seoul, Republic of Korea, 101 Daehak-Ro Jongno-Gu, Seoul, 03080, Republic of Korea Tel: +82-2-2072-4743; Fax: +82-2-762-9662

e-mail: [kauri670@empal.com](mailto:kauri670@empal.com)
