# Supplementary material for: Poor prognosis of NSCLC located in lower lobe is partly mediated by lower frequency of EGFR mutations
Source: Sci Rep. 2020 Sep 10;10:14933. doi: 10.1038/s41598-020-71996-7 (PMC7483476; doi:10.1038/s41598-020-71996-7)
Supplement: Supplementary file 2 — Supplementary Information 2. [file 41598_2020_71996_MOESM2_ESM.docx]

**Supplementary information 2. Multivariable Cox proportional hazard analysis according to tumor location in adenocarcinoma**

| **Variable** | **Model 1** | | | **Model 2** | | |
| --- | --- | --- | --- | --- | --- | --- |
|  | **HR** | **95% CI** | ***P* value** | **HR** | **95% CI** | ***P* value** |
| Tumor location, lower lobe | 1.36 | 1.12-1.65 | 0.002 | 1.22 | 0.91-1.64 | 0.019 |
| Age ≥60 year | 1.82 | 1.45-2.29 | <0.001 | 1.69 | 1.20-2.39 | 0.003 |
| Sex, male | 0.84 | 0.61-1.15 | 0.28 | 1.72 | 1.01-2.95 | 0.048 |
| Ever smoking | 1.51 | 1.10-2.07 | 0.011 | 1.98 | 1.16-3.39 | 0.013 |
| ECOG ≥2 | 2.15 | 1.63-2.84 | <0.001 | 1.74 | 1.15-2.64 | 0.009 |
| Presence of symptoms | 1.32 | 1.07-1.64 | 0.010 | 1.16 | 0.84-1.59 | 0.37 |
| BMI: 23.0-24.9 kg/m^2^ | 0.93 | 0.74-1.18 | 0.56 | 1.06 | 0.75-1.50 | 0.73 |
| BMI: ≥25.0 kg/m^2^ | 0.91 | 0.73-1.15 | 0.44 | 0.94 | 0.65-1.34 | 0.72 |
| SUV of main mass ≥ 11.2 | 1.40 | 1.15-1.70 | 0.001 | 1.28 | 0.95-1.71 | 0.10 |
| Stage II | 2.04 | 1.16-3.60 | 0.013 | 2.23 | 0.95-5.25 | 0.07 |
| Stage III | 4.45 | 2.89-6.85 | <0.001 | 5.47 | 2.79-10.73 | <0.001 |
| Stage IV | 12.06 | 8.15-17.85 | <0.001 | 13.57 | 7.14-25.81 | <0.001 |
| ALK translocation | 0.42 | 0.27-0.65 | <0.001 | 0.32 | 0.15-0.65 | 0.002 |
| Active treatment | 0.28 | 0.19-0.43 | <0.001 | 0.33 | 0.19-0.59 | <0.001 |
| NSE ≥16.3 ng/mL |  |  |  | 1.62 | 1.19-2.22 | 0.002 |
| CYFRA 21-1 ≥3.3 ng/mL |  |  |  | 1.54 | 1.12-2.12 | 0.008 |
| EGFR mutation |  |  |  | 0.49 | 0.35-0.67 | <0.001 |

ALK=anaplastic lymphoma kinase; BMI= body mass index; CYFRA=cytokeratin fragment; NSE=neuron-specific enolase; SUV=standardized uptake value

Possible mediational factors (adenocarcinoma, NSE, CYFRA 21-1, EGFR) were excluded in analysis with model 1 and included in analysis with model 2.

**Title: Poor prognosis of NSCLC located in lower lobe is partly mediated by EGFR mutations**

**Running title: prognosis in lower lobe cancer**

**Hyun Woo Lee^1*^, Young Sik Park^2*^**, Sangshin Park^3,4^, Chang-Hoon Lee^2^

^1^ Division of Pulmonary and Critical Care, Department of Internal Medicine, Seoul Metropolitan Government-Seoul National University Boramae Medical Center, Seoul, South Korea.

^2^ Division of Pulmonary and Critical Medicine, Department of Internal Medicine, Seoul National University College of Medicine, Seoul National University Hospital, Seoul, South Korea

^3^ Department of Pediatrics, Center for International Health Research, Rhode Island Hospital, The Warren Alpert Medical School of Brown University, Providence, RI, United States

^4^ Graduate School of Urban Public Health, University of Seoul, Seoul, Republic of Korea

*Two co-first authors were equally contributed to the present work.

**Corresponding author:** Chang-Hoon Lee, M.D., Associate Professor, Division of Pulmonary and Critical Care Medicine, Department of Internal Medicine, Seoul National University Hospital, Seoul, Republic of Korea, 101 Daehak-Ro Jongno-Gu, Seoul, 03080, Republic of Korea Tel: +82-2-2072-4743; Fax: +82-2-762-9662

e-mail: [kauri670@empal.com](mailto:kauri670@empal.com)
