# Supplementary material for: Poor prognosis of NSCLC located in lower lobe is partly mediated by lower frequency of EGFR mutations
Source: Sci Rep. 2020 Sep 10;10:14933. doi: 10.1038/s41598-020-71996-7 (PMC7483476; doi:10.1038/s41598-020-71996-7)
Supplement: Supplementary file 3 — Supplementary Information 3. [file 41598_2020_71996_MOESM3_ESM.docx]

**Supplementary information 3. Causal mediation analysis in lung adenocarcinoma patients**

**
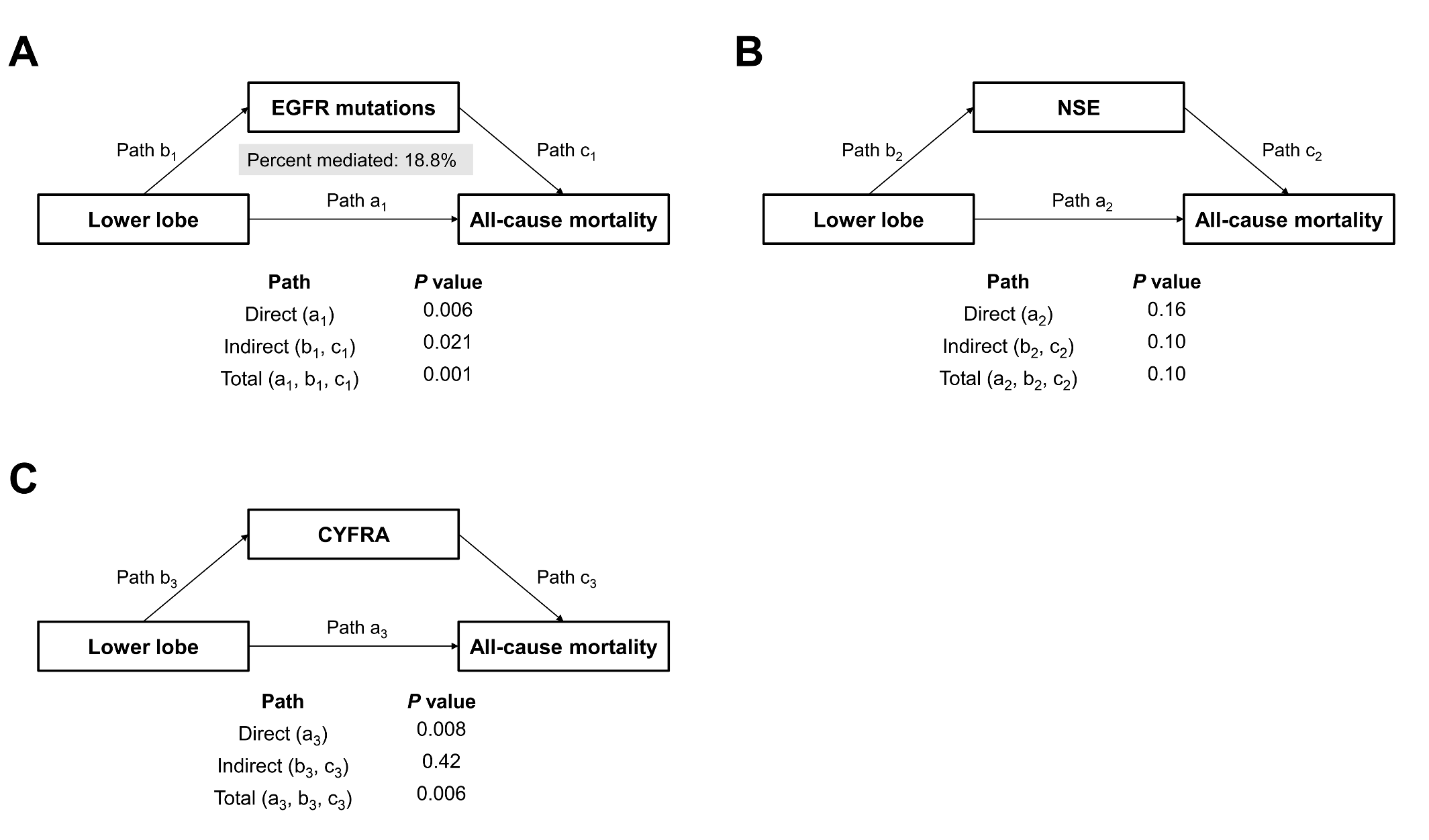
**

(A), Mediation analysis for indirect effect of EGFR mutations; (B), Mediation analysis for indirect effect of NSE; (C), Mediation analysis for indirect effect of CYFRA

CYFRA, cytokeratin fragment; EGFR, epidermal growth factor receptor mutations; NSE, neuron-specific enolase

**Title: Poor prognosis of NSCLC located in lower lobe is partly mediated by EGFR mutations**

**Running title: prognosis in lower lobe cancer**

**Hyun Woo Lee^1*^, Young Sik Park^2*^**, Sangshin Park^3,4^, Chang-Hoon Lee^2^

^1^ Division of Pulmonary and Critical Care, Department of Internal Medicine, Seoul Metropolitan Government-Seoul National University Boramae Medical Center, Seoul, South Korea.

^2^ Division of Pulmonary and Critical Medicine, Department of Internal Medicine, Seoul National University College of Medicine, Seoul National University Hospital, Seoul, South Korea

^3^ Department of Pediatrics, Center for International Health Research, Rhode Island Hospital, The Warren Alpert Medical School of Brown University, Providence, RI, United States

^4^ Graduate School of Urban Public Health, University of Seoul, Seoul, Republic of Korea

*Two co-first authors were equally contributed to the present work.

**Corresponding author:** Chang-Hoon Lee, M.D., Associate Professor, Division of Pulmonary and Critical Care Medicine, Department of Internal Medicine, Seoul National University Hospital, Seoul, Republic of Korea, 101 Daehak-Ro Jongno-Gu, Seoul, 03080, Republic of Korea Tel: +82-2-2072-4743; Fax: +82-2-762-9662

e-mail: [kauri670@empal.com](mailto:kauri670@empal.com)
